# Early Hydration Characteristics and Kinetics Model of Ordinary Portland Cement-Calcium Sulfoaluminate Cement Composites

**DOI:** 10.3390/ma18112559

**Published:** 2025-05-29

**Authors:** Jincai Chen, Bo Xie, Zhongyu Lu, Shaohua He, Shuqian Ma

**Affiliations:** School of Civil and Transportation Engineering, Guangdong University of Technology, Guangzhou 510006, China; chenjc@gdut.edu.cn (J.C.); gdutxb2326@163.com (B.X.); 18813212530@163.com (S.M.)

**Keywords:** ordinary Portland cement, calcium sulfoaluminate cement, composite cement, early hydration, hydration kinetics

## Abstract

This study investigates the early hydration characteristics and kinetics of ordinary Portland cement (OPC) and calcium sulfoaluminate cement (CSA) composite pastes. The hydration mechanisms of OPC-CSA systems with different proportions are analyzed through zonal analysis and the Krstulović–Dabić method. The experimental results show that in OPC-dominated systems, an appropriate amount of CSA promotes the rapid hydration of ye’elimite and optimizes the cumulative hydration heat and pore structure. However, excessive CSA inhibits hydration due to alkalinity imbalance. In CSA-dominated systems, 10% OPC increases the alkalinity, promoting ye’elimite to hydrate into ettringite. Higher OPC content hinders the hydration process due to ion concentration imbalance. The kinetics model indicates that CSA accelerates the interfacial reaction and diffusion in the OPC system, while OPC reduces the overall hydration rate of the CSA system. Microscopic analysis confirms that the composite system improves the pore structure through mineral interaction. In the OPC-dominated area, the pore structure is mainly composed of small and dense pores. In the CSA-dominated area, the characteristics of large pores are affected by the expansion properties of CSA and hydration heat. This study constructs a coupling mechanism of alkalinity regulation and crystal nucleus generation, providing a theoretical basis for the design of high-performance composite cement materials.

## 1. Introduction

Ordinary Portland cement (OPC) is commonly used due to its high strength, as well as its excellent resistance to frost and carbonation [1,2,3,4,5]. However, it has a relatively long setting time and poor resistance to soft water and chemical erosion. These limitations restrict its use in marine constructions and environments exposed to corrosive media [6,7,8,9,10]. Calcium sulfoaluminate cement (CSA) is a sustainable material known for its outstanding resistance to acid-alkali corrosion. Its hydration products, specifically ettringite and aluminum hydroxide gel (AH_3_), effectively protect against erosion caused by saline substances, making it well-suited for emergency repairs and marine constructions [11,12,13,14,15]. CSA has a short setting time, rapid carbonation rate, inferior long-term mechanical properties, and porous microstructure, which can lead to a decline in strength over time, posing limitations to its engineering applications. CSA shortages have been addressed by recently incorporating traditional OPC, forming an OPC-CSA composite that offers good workability and consistent strength development [16,17,18,19,20,21]. However, the performance of OPC-CSA varies significantly with the proportions of the constitutive materials, making it complex to determine the optimized compositions.

The hydration reaction is crucial for comprehending OPC-CSA performance. Free lime in OPC creates an alkalinity condition that accelerates ye’elimite hydration in CSA [22,23], where competitive reactions between OPC’s tricalcium aluminate (C_3_A) and CSA’s ye’elimite govern overall kinetics. C_3_A rapidly forms calcium aluminate hydrate, while ye’elimite undergoes sulfate-dependent hydration: in Portlandite-rich environments, it reacts with Ca(OH)_2_ and gypsum to form ettringite (AFt) at a rate 1.5–2 times faster than OPC’s C_3_S. Recent studies by Gastaldi et al. [20] and Bertola et al. [21] further demonstrated that OPC-derived calcium hydroxide promotes ye’elimite-to-ettringite hydration and improves microstructural densification in ternary OPC-CSA systems. Conversely, in CSA-dominated systems with insufficient alkalinity, ye’elimite yields aluminum hydroxide gel, compromising early strength. Portlandite addition shifts hydration toward AFt dominance, as calcium sulfate reacts with AH_3_ to form additional ettringite and reduce AH_3_ content, thereby enhancing OPC-derived phases. OPC’s alite hydrates via two pathways, forming Strätlingite or a calcium silicate–Portlandite mixed gel. In high-OPC systems, tricalcium aluminate initially forms ettringite, but a surface-coating AFt layer hinders subsequent hydration [24,25,26,27]. The ion concentration gradient (Ca^2+^, AlO_2_^−^, SO_4_^2−^) from OPC-CSA mixing ratios dictates nucleation sites and diffusion rates: a 10% OPC addition to CSA increases AFt formation by 30% via alkalinity enhancement [28]. Notably, insufficient calcium sulfate promotes ettringite-to-monosulfate transformation and ye’elimite-driven monosulfate formation, complicating long-term hydration [29].

The macroscopic performance of cementitious systems is fundamentally governed by their mineral composition, phase assemblages, and microstructural characteristics. The OPC-CSA composite system, characterized by its complex blend of constituents and intricate hydration reactions, presents significant challenges for conventional methods of hydration analysis. However, advanced approaches employing single-particle and nucleation–growth models have proven valuable in elucidating the hydration mechanisms within such systems [30,31]. Krstulović–Dabić’s work [32] identifies nucleation and crystal growth (NG), interfacial reactions (I), and diffusion (D) as the three primary mechanisms governing cement hydration. These processes occur concurrently, with the slowest acting as the rate-determining step. The Krstulović–Dabić model has been extensively employed to investigate the hydration kinetics of composite cementitious materials, particularly those incorporating high-performance water reducers, mineral admixtures, and other supplementary cementitious materials [33,34,35,36,37,38]. Given the complexity of hydration reactions in these composite systems, theoretical kinetic curves offer a more accurate representation of the hydration process. Therefore, hydration kinetics modeling holds significant promise for deciphering the hydration mechanisms of OPC-CSA composites across a range of mixing ratios, ultimately contributing to the development of advanced, high-performance cement-based materials.

This study investigates the early hydration characteristics and kinetics of OPC-CSA composite cement pastes, focusing on how mixing ratios influence hydration mechanisms. By integrating the Krstulović–Dabić kinetic model with multi-scale characterization, the research reveals the synergistic effects of OPC-derived alkalinity and CSA-induced crystal nucleation—a previously unreported mechanism in OPC-CSA systems. Isothermal calorimetry quantified heat evolution, while the model simulated kinetics to clarify temporal processes. The composition and phase assemblage of hydration products were characterized using Fourier transform infrared spectroscopy (FT-IR), X-ray diffraction (XRD), and thermogravimetric analysis (TG). Furthermore, scanning electron microscopy (SEM) coupled with energy dispersive spectrometry (EDS) was employed to analyze the microstructural morphology and determine the atomic ratios of Al/Si and S/Si within the cement matrix. This integrated approach provides insights into mix-ratio-regulated hydration pathways, offering a theoretical basis for designing high-performance composite cements with optimized early strength, dense pores, and chemical resistance—critical for sustainable infrastructure in harsh environments.

## 2. Materials and Methods

### 2.1. Experimental Materials

**Figure 1:**
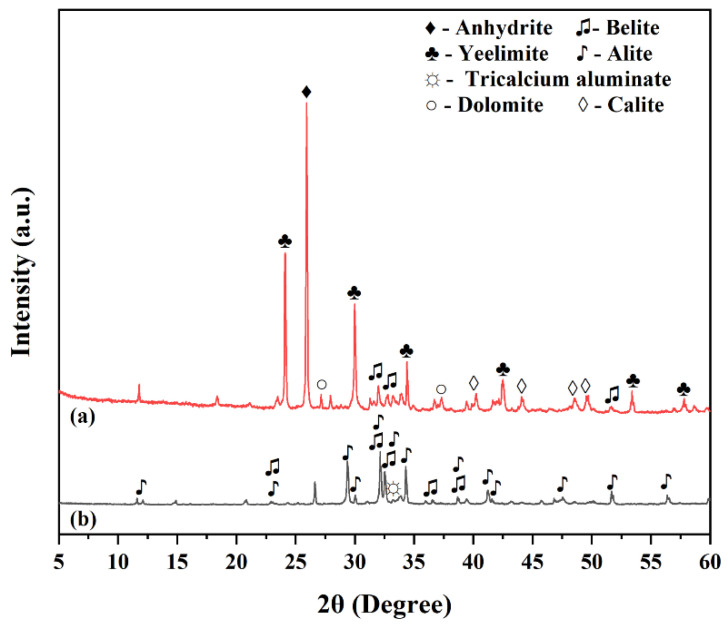
XRD results of CSA (a) and OPC (b).

This study used calcium sulfoaluminate cement (CSA 42.5) and ordinary Portland cement (OPC 42.5) as the main cementitious materials. Characterization by XRD patterns (Figure 1) showed that the main mineral components of CSA included ye’elimite, anhydrite, belite, dolomite, and calcite. In contrast, OPC mainly consisted of alite (C3S), belite (C2S), and tricalcium aluminate (C3A). Chemical compositions of OPC and CSA are presented in Table 1. The OPC contained 59.50% CaO, 18.60% SiO_2_, and 5.40% Al_2_O_3_, with minor components including Fe_2_O_3_ (2.80%), MgO (2.90%), and SO_3_ (4.80%). The CSA exhibited higher Al_2_O_3_ (27.67%) and SO_3_ (8.70%) contents, with CaO at 45.43% and SiO_2_ at 11.59%. These compositions confirm that OPC provides a higher CaO source for alkalinity, while CSA supplies abundant sulfate and aluminate for ettringite formation. A polycarboxylate-based high-performance water reducer with a water-reducing rate of 28% was added in the test. The water-to-binder ratio was fixed at 0.3, and the dosage of the water reducer was 0.2% of the total mass of the cementitious materials. The specific mix proportions are listed in Table 2.

All composite pastes were prepared using a planetary mortar mixer, Wuxi Jianyi Instrument Machinery Co., Ltd., Wuxi, China. Dry materials (OPC, CSA, gypsum) were first blended at 140 rpm for 2 min to ensure homogeneity, followed by gradual addition of water at 285 rpm for 3 min. Mixing ratios were strictly controlled using an electronic balance (accuracy ±0.1 g) to achieve the formulations in Table 2. Each mixture was prepared in triplicate to minimize experimental errors, with measurement deviations within ±1.5% for binder components and ±0.5% for water content.

The OPC-CSA system was divided into two groups: the P series with high OPC content (CSA dosages of 0%, 10%, and 20%) and the S series with high CSA content (OPC dosages of 0%, 10%, and 20%). The specimen numbering follows the format “X-ab”, where “X” indicates the series (P or S), and “ab” represents the mass percentage of CSA in the mixture. For example, P-20 refers to a mixture in the P series with a CSA content of 20%. In the S series, S-90 denotes a mixture with an OPC content of 10% (meaning a CSA content of 90%).

### 2.2. Sample Preparation

The preparation of OPC-CSA composite cement pastes followed standard experimental procedures to ensure material homogeneity and controllability of hydration reactions. A JJ-5 cement mortar mixer was used for a staged mixing process: First, OPC and CSA dry powders were pre-mixed at low speed for 30 s according to the proportions in Table 2 to ensure uniform distribution of cementitious materials. Then, a polycarboxylate superplasticizer solution (pre-dissolved in a fixed amount of water at a dosage of 0.2% by mass of the cementitious materials) was added to the mixing system at a constant flow rate. Manual homogenization for 30 s was performed to fully wet the solid-liquid interface. Finally, mechanical mixing at low speed was conducted for 1 min to complete paste preparation.

It is noted that the water-to-binder ratio was strictly controlled at 0.30 to maintain the comparability of rheological properties and microstructures. This ratio was selected based on preliminary experiments balancing workability and early strength. Specimens were cast and cured in a standard chamber, Hebei Xinmeihua Test Instrument Co., Ltd., Cangzhou, China (20 ± 1 °C, RH ≥ 95%) until the target age, then demolded and immediately immersed in absolute ethanol for 48 h to arrest hydration. To mitigate carbonation, all post-curing handling was performed under an inert atmosphere, and samples were stored in sealed conditions throughout the process.

### 2.3. Testing Methods

#### 2.3.1. Hydration Heat Analysis

Isothermal calorimetry was performed using an American TAM Air eight-channel microcalorimeter, TA Instruments, New Castle, DE, USA to analyze the hydration heat. The heat release data of cement samples were accurately recorded by an isothermal conduction calorimeter. To determine the hydration heat, the obtained data were normalized based on the mass of the tested samples. Then, the hydration heat release rate was calculated by differentiating the hydration heat results. These data were later used in the Krstulović–Dabić hydration kinetics model described in Section 2.4 to derive parameters such as reaction rate constants.

The entire test was conducted under strict temperature control of 20 ± 0.02 °C for 72 h to ensure accurate monitoring of the early hydration heat release behavior (0–72 h) of cement pastes. Combined with the Knudsen equations (Equations (7)–(9)) in Section 2.4, the theoretically maximum heat release (Q_max_) and hydration degree (α) were precisely converted from the experimentally measured hydration heat (Q_t_). The specific conversion method is detailed in the kinetics model section, and only the basic data processing steps for hydration heat analysis are described here.

#### 2.3.2. Phase Analysis

XRD analysis was performed using a Bruker D8 Advance X-ray diffractometer (Bruker, Karlsruhe, Germany) to characterize the crystal structure of samples. The instrument was equipped with a copper target, operating at a voltage of 50 kV and a current of 60 mA. Carefully prepared powder samples were tested with a scanning rate of 5° per minute in the 2θ range of 5° to 60°. By analyzing the diffraction patterns, the phase composition of unhydrated minerals (such as ye’elimite, calcite, anhydrite, tricalcium aluminate, belite, and dolomite) and key hydration products (such as ettringite and calcium hydroxide) in the cement pastes was identified.

For samples hydrated for 3 days, an American Thermo Fisher iN10 Fourier Transform Infrared Spectrometer (FT-IR), Thermo Fisher Scientific, Waltham, MA, USA was used for testing. Before the test, about 0.001 g of sample powder was mixed and ground with 0.15 g of potassium bromide and then pressed into a thin slice. Subsequently, it was scanned in the wavenumber range of 400–4000 cm^−1^. By analyzing the absorption peaks (such as the OH^−^ stretching vibration peak at 3422 cm^−1^, the [SO_4_] stretching vibration peak of ettringite at 1111 cm^−1^, and the asymmetric stretching vibration peak of the Si-O bond in C-S-H near 970 cm^−1^), the functional group information of the hydration products was obtained, providing supplementary evidence for the phase composition analysis.

A PerkinElmer TGA 4000 thermogravimetric analyzer (PerkinElmer, Waltham, MA, USA) was used to measure the TG curves of 3-day-hydrated sample powders. DTG curves were derived simultaneously to identify and quantify the main hydration products and the proportion of bound water. During the test, samples were heated from room temperature to 900 °C at a constant rate of 10 °C/min and decomposed under a continuous nitrogen flow of 100 mL/min. Based on the characteristics of endothermic peaks in DTG curves within temperature ranges 50–150 °C (ettringite lattice dehydration), 150–250 °C (monosulfate dehydration), 400–500 °C (calcium hydroxide dehydration), and 600–750 °C (calcite decarbonation) and combined with TG data, the composition of hydration products was further analyzed.

#### 2.3.3. Microstructural and Pore Structure Analysis

SEM using a Hitachi S-3400 N-II (Hitachi High-Tech Science Corporation, Tokyo, Japan) was employed to analyze the microstructure of samples. The acceleration voltage of the instrument was set at 15 kV. Before testing, all samples were coated with a thin gold layer under vacuum to improve conductivity and ensure imaging quality. Sample preparation involved cutting hydrated pastes into 5 mm cubes, polishing their surfaces to expose fresh microstructures, and drying at 60 °C for 24 h to remove residual moisture. Sputter coating with a thin gold layer (approximately 10 nm thickness) was performed under vacuum to enhance conductivity. Imaging was conducted using secondary electrons at an acceleration voltage of 15 kV to visualize surface morphology, with backscattered electrons occasionally used for elemental contrast when analyzing phase distributions. SEM observations provided visual evidence of the morphology of hydration products (e.g., the shape and distribution of C-S-H gel and ettringite crystals) and microstructural evolution (e.g., the distribution of unhydrated particles, pores, and cracks), supporting the analysis of hydration processes and product interactions.

EDS was used to quantitatively analyze the atomic concentration ratios of Al, S, and Si in the cement matrix. By detecting elemental signals in specific regions, Al/Si and S/Si atomic ratio data were obtained to reflect the hydration progress of OPC and CSA and elemental distribution characteristics at different hydration ages.

Pore structure analysis was conducted using an AutoPore V 9620 mercury porosimeter (Micromeritics, Norcross, GA, USA), which accurately measured the pore size distribution and total pore volume of samples. During sample preparation, hardened pastes were cut into approximately 5 mm-sized specimens and dried in an oven at 60 °C for 24 h to remove moisture, ensuring consistent testing conditions. The measurement range covered 5 nm to 800 μm, capturing pore characteristics across different scales and providing data support for evaluating pore structure parameters (e.g., most probable pore diameter, cumulative pore volume) of the OPC-CSA composite system.

All experimental methods, materials used, unique characteristics, and key parameters are detailed in Table 3.

### 2.4. Hydration Kinetic Model

Based on isothermal calorimetry results, the Krstulović–Dabić model [32] was used to develop early-stage kinetic models for OPC-CSA composite cements with different mix ratios. The model divides the complex hydration reaction system into three basic processes: nucleation and growth (NG), interfacial reaction (I), and diffusion (D). Kinetic equations describing the relationship between hydration degree (α) and reaction time (t) were established for each process.

For the NG process, it is assumed that the formation and growth of hydration product nuclei follow a power-law pattern. The corresponding kinetic equation is as follows:

(1) $${\left[-\ln\left(1-\alpha \right)\right]}^{1/n}=K_{1}\left(t-t_{0}\right)=k_{1}^{\prime }\left(t-t_{0}\right)$$

The I process describes the interfacial chemical reaction between cement particles and water, described by the equation:

(2) $$1-{\left(1-\alpha \right)}^{1/3}=K_{2}r^{-1}\left(t-t_{0}\right)=k_{2}^{\prime }\left(t-t_{0}\right)$$

The D process accounts for the hindrance of ion diffusion by the hydration product layer with the equation:

(3) $${\left[1-{\left(1-\alpha \right)}^{1/3}\right]}^{2}=K_{3}r^{-2}\left(t-t_{0}\right)=k_{3}^{\prime }\left(t-t_{0}\right)$$

Differentiating these equations yields the reaction kinetics (reaction rates) for NG, I, and D processes:

For process NG:

(4) $$d\alpha /dt=F_{1}\left(\alpha \right)=k_{1}^{\prime }n\left(1-\alpha \right){\left[-\ln\left(1-\alpha \right)\right]}^{\left(n-1\right)/n}$$

For process I:

(5) $$d\alpha /dt=F_{2}\left(\alpha \right)=k_{2}^{\prime }3{\left(1-\alpha \right)}^{2/3}$$

For process D:

(6) $$d\alpha /dt=F_{3}\left(\alpha \right)=k_{3}^{\prime }3{\left(1-\alpha \right)}^{2/3}/\left[2-2{\left(1-\alpha \right)}^{1/3}\right]$$

In these equations, α is the hydration degree of the sample; $K_{x}$ and $k_{x}^{\prime }$ are the reaction rate constants for the three processes; t and $t_{0}$ are the hydration time and induction period end time, respectively; *n* is the crystal growth constant; and *r* is the radius of reacting particles.

The theoretical maximum heat release (*Q_max_*) and hydration degree (*α*) were converted from experimentally measured hydration heat (*Q_t_*) using equations by Kundsen [39], as follows [40,41]:

(7) $$1/Q_{t}=1/Q_{max}+t_{50}/\left[Q_{max}\left(t-t_{0}\right)\right]$$

(8) $$\alpha \left(t\right)=Q\left(t\right)/Q_{max}$$

(9) $$d\alpha /dt=d\left(Q\left(t\right)/Q_{max}\right)/dt=\left(1/Q_{max}\right)*dQ/dt$$

Here, *t_50_* is the time when 50% of the theoretical maximum heat is released.

To ensure simulation accuracy, the least-squares method [32] was used to minimize the standard deviation between experimental values and fitted results, with fittings determined by linear regression.

## 3. Results and Discussion

### 3.1. Hydration Heat Evolution Characteristics

#### Failure Pattern

**Figure 2:**
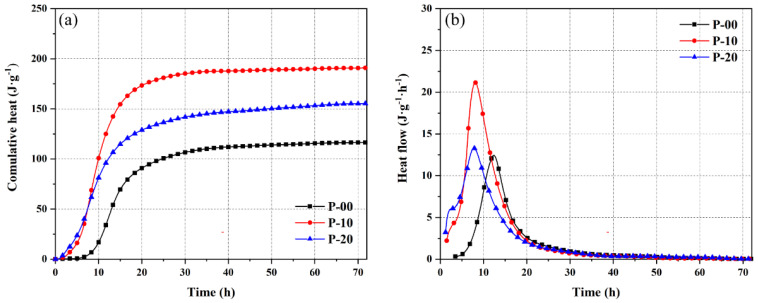
Cumulative heat (**a**) and heat flow (**b**) of OPC-CSA blends in the P area.

**Figure 3:**
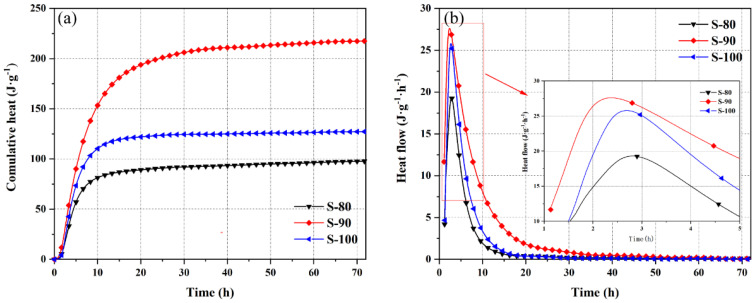
Cumulative heat (**a**) and heat flow (**b**) of OPC-CSA blends in the S area.

Figure 2 and Figure 3 show the hydration heat evolution processes of OPC-CSA composite cement pastes in the P area (high-OPC content systems) and S area (high-CSA content systems). Figure 2a and Figure 3a present cumulative hydration heat curves, while Figure 2b and Figure 3b display heat release rate curves. These curves provide key data support for analyzing the hydration kinetics characteristics of the two areas.

In the P area, as the CSA content increases, the cumulative hydration heat and peak heat release rate of the system show a trend of first increasing and then decreasing. For example, at the 3-day hydration age, the cumulative hydration heats of P-10 and P-20 are 190.9 J/g and 155.4 J/g, respectively. These values represent increases of 63.8% and 33.4% compared to pure OPC (P-00, 116.5 J/g), indicating that an appropriate amount of CSA (10%) significantly promotes the overall hydration degree of the cementitious materials.

The heat release rate curves show that the peak heat release rates of P-10 and P-20 during the hydration acceleration period are higher than that of P-00, with peak times advancing by 4.3 h and 4.6 h, respectively, compared to P-00. Notably, the heat release rate of P-10 exceeds that of P-20 after 5.5 h and reaches the highest peak. The transitional characteristics of P-20 in the acceleration stage suggest a connection effect between the rapid hydration of CSA and the initial hydration stage of OPC (details of related hydration product transformation and microstructural effects are provided in Section 3.2 and Section 3.3).

The hydration heat release reactions in the S area exhibit different characteristics from those in the P area. The peak heat release rate appears within the first 2–3 h of hydration, which is faster than in the P area. For S-90 (containing 10% OPC), the cumulative hydration heat at 3 days is significantly increased by 71.0% compared to pure CSA (S-100), indicating that a small amount of OPC effectively improves the hydration degree of the high-CSA system.

Comparing the heat release rate curves, the peak heat release rate of S-90 is higher than that of S-100 and occurs earlier, while the peak heat release rate of S-80 (containing 20% OPC) is lower and delayed. During the heat release deceleration stage, the heat release rate of S-90 remains at a high level for approximately 32 h, significantly longer than the 15 h observed in S-80 and S-100. This reflects the sustained promoting effect of 10% OPC on the hydration process in the S area (the influence of free lime introduced by OPC on the CSA hydration pathway is detailed in the XRD analysis of Section 3.2.1).

The differences in hydration heat evolution between the P and S areas are closely related to the reaction sequence of cementitious materials, distribution of crystallization nucleation sites, and changes in ion concentration. In the P area, the ye’elimite in CSA undergoes rapid hydration in the calcium hydroxide environment provided by OPC. This accelerates the formation of ettringite and promotes the hydration of tricalcium aluminate and belite in OPC. In the S area, calcium hydroxide introduced by OPC provides an alkaline condition for ye’elimite hydration, optimizing the early hydration pathway. This aligns with Gastaldi et al. [20], who observed that OPC’s high CaO content enhances AFt formation in CSA-dominated pastes through alkalinity regulation.

The heat release characteristics under different mix ratios provide macrothermal behavior evidence for subsequent analysis of hydration products (Section 3.2), observation of microstructures (Section 3.3), and construction of the kinetics model (Section 3.4).

### 3.2. Hydration Product Composition and Phase Evolution

#### 3.2.1. XRD Analysis

**Figure 4:**
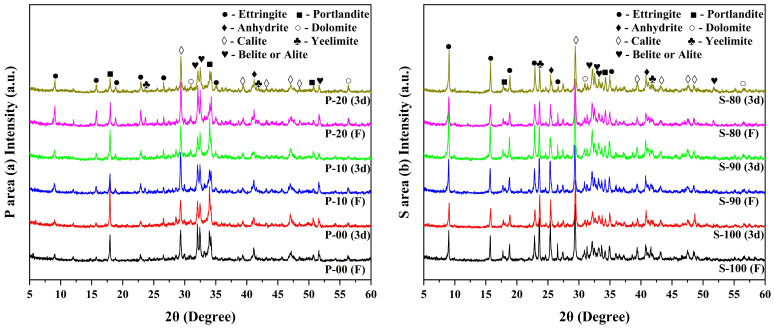
XRD patterns of OPC-CSA blends in the P area (**a**) and S area (**b**) at peak heat flow (F) and 3d curing age (3d).

Figure 4 shows the XRD patterns of OPC-CSA composite cement pastes at the peak heat release rate (F) and 3-day curing age (3d). By comparing the mineral composition of raw materials (XRD patterns of CSA and OPC shown in Figure 1), the residual characteristics of unhydrated minerals and the evolution of crystalline hydration products under different mix ratios are revealed. Unhydrated minerals in the paste include ye’elimite (a main mineral of CSA), calcite, anhydrite, tricalcium aluminate, belite, and dolomite. As the main crystalline hydration products, ettringite (AFt) and calcium hydroxide (CH) show changes in diffraction peak intensities that directly reflect differences in the hydration process. Due to the amorphous nature of C-S-H gel and aluminum hydroxide gel (AH_3_), their microstructures do not produce characteristic diffraction signals in the patterns [42].

In the P area, the diffraction peaks of ye’elimite in P-10 and P-20 are significantly weakened during the peak heat release stage and disappear completely after 3 days of hydration. This indicates that ye’elimite in CSA undergoes rapid hydration in the CH-rich alkaline environment provided by OPC hydration. This phenomenon is closely related to the hydration pathway of ye’elimite: when sufficient CH exists in the system (e.g., in P-10 and P-20), ye’elimite hydration primarily involves reactions that consume CH, generating a large amount of AFt and promoting the hydration of tricalcium aluminate in OPC. However, the intensity of the AFt diffraction peak in P-20 decreases at 3 days of hydration compared to the peak heat release stage. This suggests that part of the AFt transforms into monosulfate (AFm) due to the coating of mineral surfaces by a hydration product layer (e.g., dense deposition of AFt crystals) under low water-to-binder ratio conditions, which is consistent with the mechanism of “ettringite layer covering hindering subsequent hydration” described in the literature [24]. Notably, the intensity of the CH diffraction peak in P-20 is significantly reduced at 3 days of hydration. Combined with the result that its cumulative hydration heat is lower than that of P-10 (Section 3.1), it is inferred that ion concentration imbalance caused by excessive CSA (e.g., excessive sulfate ions) may inhibit the continuous hydration of tricalcium aluminate and belite in OPC, thereby reducing CH production.

In the S area, the incorporation of OPC increases the alkalinity of the system by introducing free lime, causing the early hydration pathway of ye’elimite to shift from “direct hydration without CH participation” (mainly generating AH_3_ and a small amount of AFt) to “CH-induced alkaline hydration” (preferentially generating AFt). This is manifested by the significantly higher AFt diffraction peak intensities in S-90 and S-80 at 3 days of hydration compared to pure CSA (S-100). Although the CSA contents in S-80 and S-90 are lower than that in S-100, the catalytic effect of CH before the peak heat release makes the AFt production amounts in the three similar, reflecting the regulatory effect of alkalinity on the hydration pathway of ye’elimite. However, the peak heat release rate of S-80 is lower than that of S-90 (Figure 3b), which may be related to the reduced reaction sites and decreased ion diffusion efficiency caused by the lower CSA content in this mix ratio.

In addition, the diffraction peaks of tricalcium aluminate and belite in the S area samples did not significantly weaken at 3 days of hydration, indicating that the silicate minerals in OPC have a relatively low early hydration degree in the high-CSA environment [28]. Since tricalcium aluminate is the main mineral in OPC that generates CH, the strong signal of its diffraction peak indirectly reflects the hydration inhibition of this mineral in the high-CSA system, resulting in a limited supply of CH in the system. This forms a logical correspondence with the dependence of the ye’elimite hydration pathway on alkalinity—although OPC introduces a potential source of CH, its own mineral activity is inhibited in high-CSA mix ratios, so that the actual amount of CH participating in ye’elimite hydration can only meet the early catalytic demand. Combining the evolution of the diffraction peaks of ye’elimite and AFt, it can be seen that the CH introduced by OPC effectively promotes the AFt-type hydration of ye’elimite in the S area by changing the local chemical environment. However, an excessively high OPC content (such as S-80) may have a negative impact on the sustainability of the hydration process due to the steric hindrance between mineral particles and ion concentration imbalance.

#### 3.2.2. FT-IR Analysis

**Figure 5:**
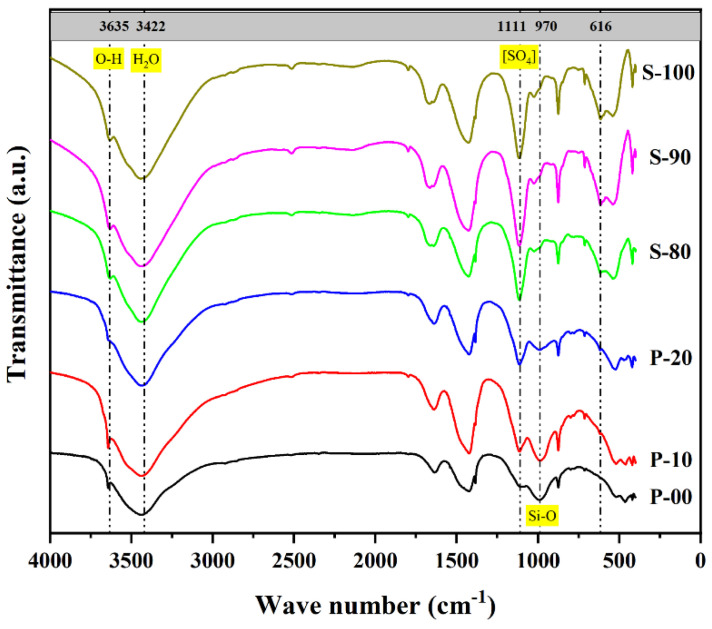
FT-IR spectrum of OPC-CSA blends at 3d curing age.

Figure 5 shows the FT-IR spectra of OPC-CSA composite cement pastes after 3 days of hydration. Changes in the intensity and position of characteristic absorption peaks reveal the evolution of functional groups and corresponding differences in hydration processes under different mix ratios. The broad peak at 3422 cm^−1^ is attributed to the stretching vibration of OH^−^. Its intensity reflects the total content of hydration products in the system, such as CH, AFt, and C-S-H gel, which is consistent with the trend of cumulative hydration heat in the hydration heat analysis (Section 3.1). The strong absorption band at 1111 cm^−1^ corresponds to the stretching vibration of [SO_4_]^2−^ in the ettringite (AFt) structure. The weak absorption peak at 3635 cm^−1^ is the O-H vibration of AFt crystalline water, while the absorption band near 970 cm^−1^ originates from the asymmetric stretching vibration of Si-O bonds in C-S-H gel. This band serves as a key indicator for evaluating the hydration degree of tricalcium aluminate and belite in OPC [43]. The absorption peak at 616 cm^−1^ is caused by the vibrational coupling of [AlO_4_] and [SO_4_] tetrahedral units in ye’elimite, and its intensity change directly reflects the hydration state of the main mineral in CSA.

In the P area, as the CSA content increases (P-00 → P-10 → P-20), the intensity of the characteristic peak for AFt at 1111 cm^−1^ shows an increasing trend, while the absorption peak of ye’elimite at 616 cm^−1^ does not significantly weaken. This indicates that ye’elimite in CSA preferentially hydrates in the CH-rich environment provided by OPC without obvious inhibition, which is consistent with the rapid disappearance of ye’elimite diffraction peaks in P-10 and P-20 observed in the XRD analysis (Section 3.2.1). Compared with P-00, the intensity of the Si-O absorption band at 970 cm^−1^ in P-10 is significantly enhanced, indicating a higher hydration degree of tricalcium aluminate and belite in OPC and the generation of more C-S-H gel. This benefits from the promoting effect of sulfate ions introduced by CSA on OPC hydration. However, the intensity of the Si-O absorption band in P-20 decreases compared to P-10, suggesting that excessive CSA may inhibit the hydration of OPC silicate minerals. This finding aligns with the results that P-20 has lower cumulative hydration heat than P-10 (Section 3.1) and weaker CH diffraction peaks (Section 3.2.1), indicating that a 10% CSA content is the threshold for optimizing the hydration performance in the P area.

In the S area, the intensity of the ye’elimite characteristic peak (616 cm^−1^) in S-90 and S-80 is lower than that in S-100. This indicates that free lime introduced by OPC promotes ye’elimite hydration by increasing the system alkalinity. Notably, the Si-O absorption peak at 970 cm^−1^ in S area samples does not significantly strengthen with increasing OPC content. This peak corresponds to the vibration of Si-O bonds in C-S-H gel, and its stable intensity reflects a relatively low hydration rate of tricalcium aluminate (the main mineral in OPC that generates C-S-H through hydration) in the high-CSA environment. This is consistent with the XRD analysis in Section 3.2.1, where the diffraction peaks of tricalcium aluminate in the S area did not significantly weaken. The strong diffraction signal of tricalcium aluminate’s high crystallinity indicates insufficient hydration in high-CSA mix ratios, indirectly suggesting that the activity of OPC silicate minerals is inhibited.

Additionally, the intensity of the 1111 cm^−1^ peak (stretching vibration of [SO_4_] in ettringite AFt) in S-90 and S-80 is higher than that in S-100. This further confirms that the alkalinity introduced by OPC promotes the hydration pathway of ye’elimite toward AFt, complementing the evolution of AFt diffraction peaks observed in the XRD analysis of Section 3.2.1. The quantitative analysis of functional groups by FT-IR spectroscopy supplements XRD phase analysis: the former’s sensitivity to amorphous products (such as AH_3_ gel) compensates for the latter’s limitation of only detecting crystalline phases. Together, they reveal differences in hydration products regulated by alkalinity: in the S area, ye’elimite preferentially generates AFt under high-alkali conditions, while the limited hydration of OPC silicate minerals results in no significant increase in C-S-H gel formation.

#### 3.2.3. TG-DTG Analysis

**Figure 6:**
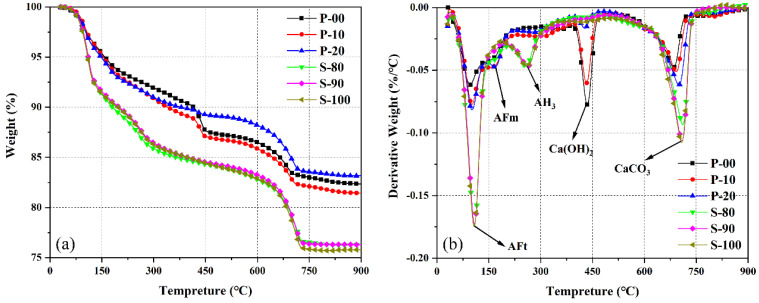
TG (**a**) and DTG (**b**) curves of OPC-CSA blends at 3d curing age.

Figure 6 shows the TG-DTG curves of OPC-CSA composite cement pastes after 3 days of hydration. Through characteristic endothermic peaks in TG and DTG curves, the composition and content changes of main hydration products are quantitatively analyzed. The endothermic peaks in the DTG curves at 50–150 °C, 150–250 °C, 400–500 °C, and 600–750 °C correspond to the lattice dehydration of ettringite (AFt), dehydration of monosulfate (AFm), decomposition of calcium hydroxide (CH), and decarbonation of calcite, respectively [44,45]. Notably, an additional endothermic peak in the 250–280 °C range for S area samples is attributed to the dehydration of aluminum hydroxide gel (AH_3_), reflecting differences in the hydration pathways of CSA under different alkalinity conditions [29].

By applying Equations (10)–(12) derived from TG curves [26,46], the contents of ettringite, AH_3_, and Ca(OH)_2_ can be accurately calculated. The specific calculation results are summarized in Table 4.

(10) $$AFt\;\left(\%\right)=\left(\frac{M_{50}-M_{150}}{M_{total}}\right)/0.35$$

(11) $${AH}_{3}\;\left(\%\right)=\left(\frac{M_{250}-M_{280}}{M_{total}}\right)*\frac{{2M}_{{AH}_{3}}}{3M_{H_{2}O}}$$

(12) $${Ca(OH)}_{2}\;\left(\%\right)=\left(\frac{M_{400}-M_{650}}{M_{total}}\right)*\frac{M_{{Ca(OH)}_{2}}}{M_{H_{2}O}}$$

wherein $M_{{AH}_{3}}$ and $M_{{Ca(OH)}_{2}}$ represent the molar masses of AH_3_ and Ca(OH)_2_, respectively, with the unit of g/mol.

The hydration product contents calculated from TG data (Table 4) show that in the P area, as the CSA content increases (P-00 → P-10 → P-20), the CH content decreases from 12.65 wt% to 9.68 wt% and then to 3.19 wt%, while the AFt content increases from 12.76 wt% to 13.67 wt% and then to 14.26 wt%. This trend is consistent with the weakening of the CH diffraction peak and the dynamic evolution of the AFt peak in XRD, indicating that ye’elimite introduced by CSA preferentially undergoes “CH-participating” hydration reactions in the CH-rich environment, consuming CH in the system and generating more AFt. The increase in AFm content in P-10 and P-20 compared to P-00 suggests that under low water-to-binder ratio conditions, part of the AFt is transformed into AFm due to changes in the reaction environment, which is consistent with the mechanism of the decrease in the AFt peak intensity during 3 days of hydration in XRD.

In the S area, the DTG curve of the pure CSA sample S-100 does not show an AFm endothermic peak, indicating that its ye’elimite hydration mainly follows the “CH-free” pathway, mainly generating AFt and AH_3_ (the 250–280 °C peak). The incorporation of OPC (S-90, S-80) introduces CH, promoting the hydration pathway to shift towards the “CH-induced” type, manifested as significantly higher AFt content than S-100 (e.g., AFt in S-90 is 23.89 wt% and in S-100 is 24.36 wt%, with the difference due to test errors and reaction kinetics differences) and a slight increase in AH_3_ content. Notably, the AFt content in S-80 (24.78 wt%) is slightly higher than that in S-90, which may be related to the more abundant CH provided by the higher OPC content in this mix ratio. However, the CH content in S-80 (calculated by TG) does not increase significantly, reflecting that CH in the S area mainly comes from the free lime of OPC rather than being generated by hydration, which is consistent with the extremely weak CH diffraction peak in the S area in the XRD analysis of Section 3.2.1.

TG-DTG analysis quantifies the composition ratio of hydration products through thermal weight loss, providing a quantitative supplement to XRD and FT-IR analysis: for the amorphous product AH_3_, its clear identification of the dehydration signal in the DTG curve compensates for the limitation of XRD in detecting non-crystalline phases, and the accurate calculation of CH content (Equations (10)–(12)) and the change in the CH diffraction peak intensity in XRD mutually confirm the data. The relationship between the increase and decrease of CH and AFt in the P area reveals the dependence mechanism of ye’elimite hydration on the OPC alkaline environment; the existence of AH_3_ and the change in AFt content in the S area reflect the transformation of the hydration pathway of ye’elimite from the “AH_3_ type” to the “AFt type” under the regulation of alkalinity. These results together indicate that the interaction between OPC and CSA significantly affects the types, contents, and formation pathways of hydration products by changing the local chemical environment (such as CH concentration and sulfate ion activity), thereby regulating the early hydration process of the composite system.

### 3.3. Microstructural and Pore Structure Characteristics

#### 3.3.1. SEM Observations and EDS Analysis

**Figure 7:**
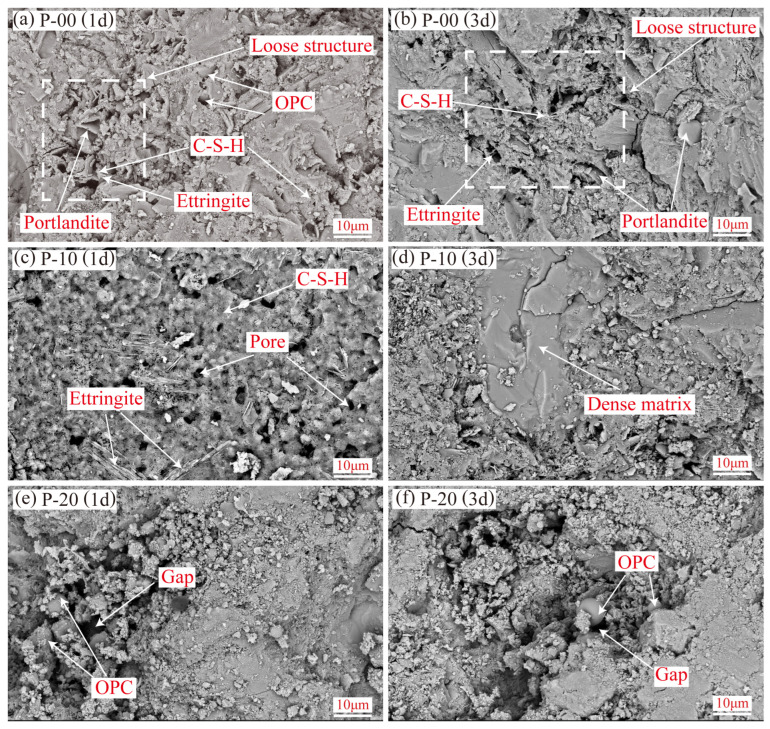
Microstructure of OPC-CSA blends in the P area at 1d (**left**) and 3d (**right**) curing ages. From top to bottom on each row are the following: P-00, P-10, and P-20.

**Figure 8:**
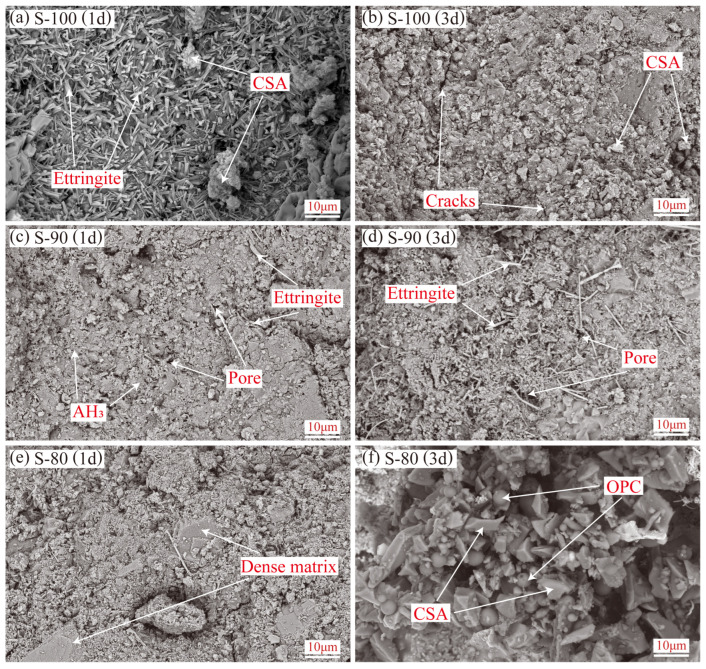
Microstructure of OPC-CSA blends in the S area at 1d (**left**) and 3d (**right**) curing age. From top to bottom on each row are the following: S-100, S-90 and s-80.

**Figure 9:**
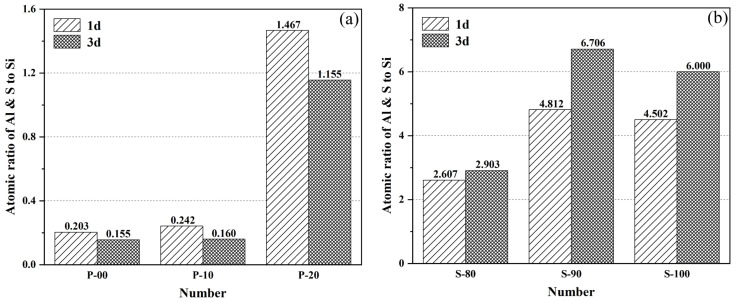
Results of atomic concentration ratio of Al and S to Si in the P area (**a**) and S area (**b**) of the cement matrix by EDS tests.

Figure 7 and Figure 8 show the microstructural characteristics (magnification of 5000×) of OPC-CSA composite cement pastes at 1-day and 3-day hydration ages. Combined with Al/Si and S/Si atomic concentration ratios from EDS tests (Figure 9), these reveal the morphology and distribution of hydration products and element migration patterns under different mix ratios.

As shown in Figure 7a, the pure OPC sample (P-00) at 1-day hydration forms a relatively loose structure where hydration products—calcium hydroxide, C-S-H gel, and a small amount of short acicular ettringite crystals—interweave. Unhydrated OPC particles are visible in the pores of the cement matrix. After 3 days of hydration (Figure 7b), although loose regions decrease, residual loose structures remain clear. This is due to the slow hydration kinetics of some OPC particles: as C-S-H gel gradually coats clinker particles, the overall hydration rate slows [47]. Notably, unhydrated particles are wrapped by the hydration product matrix after 3 days, reflecting the gradual hydration characteristics of minerals in the single OPC system.

The P-10 sample with 10% CSA exhibits significantly different micromorphology: at 1-day hydration (Figure 7c), the content and uniformity of C-S-H gel distribution increase notably, forming a continuous cementitious matrix. At 3 days (Figure 7d), C-S-H gel widely diffuses and fills pores, leading to highly densified microstructures with no obvious unhydrated particles observed. Ettringite appears as slender acicular crystals [48], consistent with the higher cumulative hydration heat (Figure 2a) and rapid hydration rate (Figure 2b) of this mix ratio. Sufficient formation of hydration products limits the growth space of ettringite, forcing it to extend in a one-dimensional direction. This structural feature indicates that moderate CSA optimizes the generation efficiency of C-S-H gel and the crystal growth environment by promoting the hydration of tricalcium aluminate and belite in OPC.

However, the microstructures of P-20 at both 1-day and 3-day hydration (Figure 7e,f) show numerous residual unhydrated OPC particles with obvious interparticle voids. This phenomenon aligns with the weakened calcium hydroxide diffraction peaks of P-20 in the XRD analysis (Section 3.2.1), indicating that excessive CSA inhibits OPC hydration, resulting in insufficient hydration product formation to effectively fill pores and forming a loose structure.

The S area exhibits different microstructural characteristics: at 1-day hydration, the pure CSA sample (S-100) generates a large number of short rod-shaped ettringite crystals, and partially hydrated CSA particles are covered by hydration products (Figure 8a). After 3 days, although the microstructure is denser, numerous cracks are still visible on the matrix surface (Figure 8b), which is consistent with relevant research results of pure CSA [49], reflecting the volume expansion and loose structure characteristics caused by the rapid hydration of CSA.

In the S-90 sample with 10% OPC, ettringite is still mainly short rod-shaped at 1 day, with a large amount of AH_3_ gel on the surface (Figure 8c). After 3 days, some ettringite crystals elongate (Figure 8d), indicating that the alkalinity introduced by OPC promotes the transformation of ye’elimite into the AFt-type hydration pathway, which is consistent with the enhanced AFt diffraction peaks in the XRD analysis (Section 3.2.1). Notably, the surface cracks in S-90 are fewer than those in S-100, but due to the influence of excessive hydration heat, there are still a large number of pores inside the matrix [50], revealing the significant impact of hydration heat on the compactness of the microstructure.

The S-80 sample forms a dense matrix at 1 day, with a large amount of hydration products covering the surface (Figure 8e), reflecting its fastest initial hydration rate, which confirms the hypothesis that the increase in OPC content promotes the formation of calcium hydroxide and accelerates the hydration of CSA. However, after 3 days, a large number of unhydrated OPC and CSA particles are still visible in the pores (Figure 8f), indicating that the early rapidly formed hydration products may hinder the subsequent diffusion of water and ions, resulting in the stagnation of the hydration process, which corresponds to the characteristics of S-80 with a lower and delayed peak heat release rate in Section 3.1.

Figure 9 presents the atomic concentration ratios of Al and S elements relative to Si in the cement matrix through EDS analysis. These ratios, as quantitative indicators, effectively reflect the differences in the hydration processes of OPC and CSA at different hydration ages. In the P area (Figure 9a), the Al/Si and S/Si atomic concentration ratios gradually decrease with the extension of hydration time. This is because after the initial rapid hydration of CSA, silicate minerals such as tricalcium aluminate and belite in OPC gradually hydrate over a longer period, resulting in a gradual increase in the content of the Si element with the formation of C-S-H gel, thereby causing the ratios to decline. Among them, the element ratios of P-10 change less compared with P-00, indicating that the 10% CSA content significantly increases the content of the Si element in the matrix by promoting OPC hydration and optimizes the hydration efficiency of silicate minerals. On the contrary, the ratios of P-20 increase significantly, reflecting that the initial rapid hydration of excessive CSA may hinder the subsequent hydration process of OPC, resulting in limited growth of Si element content. Comparing the 1-day and 3-day ages, the element ratios of P-00, P-10, and P-20 decrease by 23.61%, 34.12%, and 21.25%, respectively, and P-10 has the largest decrease, which is consistent with the SEM observation result that P-10 is the most fully hydrated and has the densest structure, indicating that the hydration degree of OPC is the highest in this mix ratio.

In the S area (Figure 9b), the element ratios show an upward trend with the hydration of CSA, reflecting the relative enrichment of aluminum and sulfur elements contained in ye’elimite and anhydrite in CSA in the matrix. The element ratios of S-90 are the highest at 1-day hydration, with an increase of 48.93% at 3 days, indicating that the hydration of CSA in S-90 is continuously promoted by the alkalinity introduced by OPC, which is consistent with the phenomenon that the ettringite crystals in S-90 elongate and the hydration degree increases with the increase of age in SEM. The increase ratio of S-80 is only 11.35%, suggesting that the hydration of CSA in S-80 is inhibited in the later stage, which forms a kinetic correlation with the observation result of a large number of unhydrated particles remaining in the pores of S-80 in SEM, indicating that an excessively high OPC content may affect the continuous hydration of CSA due to ion concentration imbalance or steric hindrance. The dynamic evolution of EDS element ratios provides quantitative support for microstructural analysis: in the P area, the hydration of OPC is optimized through the regulation of CSA, resulting in the enrichment of the Si element and the formation of a dense structure; in the S area, due to the difference in alkalinity, the promotion and inhibition of CSA hydration are differentiated, and the enrichment degree of aluminum and sulfur elements directly reflects the difference in the hydration path of ye’elimite affected by OPC. The mutual verification between this element distribution characteristic and the micro morphology further reveals the differential regulation mechanism of the mineral interaction on the hydration process in the OPC-CSA system, providing key evidence for understanding the hydration mechanism of the composite system from the perspective of element migration.

#### 3.3.2. MIP Analysis

**Figure 10:**
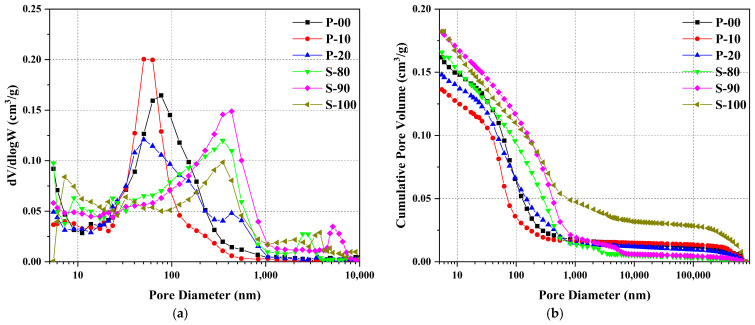
Pore size distribution (**a**) and cumulative pore volume (**b**) of OPC-CSA at 3d curing age.

Figure 10 reveals the pore structure characteristics of OPC-CSA pastes after 3 days of hydration using mercury intrusion porosimetry (MIP), AutoPore V 9620 mercury porosimeter (Micromeritics Instrument Corporation, Norcross, GA, USA). This complements the microstructural observations from SEM/EDS in Section 3.3.1, further analyzing the influence of mineral interactions on pore structure from the perspectives of pore size distribution and pore volume quantification.

As shown in Figure 10a, the P area and S area exhibit distinct pore size distribution characteristics: the most probable pore size of P area samples is concentrated below 100 nm, reflecting a relatively uniform distribution of small pores, while the characteristic peak of the S area is in the range of 340–440 nm, and a weak macropore peak appears at 2000–8000 nm. This difference is closely related to the hydration characteristics of the two types of cement: CSA rapidly generates ettringite crystals during the early stage of hydration and forms a rigid skeleton, and the accompanying volume expansion causes the structural wall to be squeezed and cracked, thereby forming macropores and microcracks [51,52,53,54], which is consistent with the cracks on the surface of the S-100 matrix (Figure 8b) and the porous structure inside S-90 (Figure 8d) observed by SEM in Section 3.3.1; in the P area, hydration products (such as C-S-H gel and slender ettringite crystals) are uniformly stacked to fill pores, effectively suppressing the development of macropores.

In the P area, with the increase of CSA content, the cumulative pore volume decreases significantly (Figure 10b), and the pore volume of P-10 with 10% content is the smallest. This phenomenon is consistent with the results of the highest cumulative hydration heat of P-10 in the hydration heat analysis in Section 3.1 and the optimized production of calcium hydroxide and AFt in XRD in Section 3.2—an appropriate amount of CSA promotes the hydration of OPC to generate more cementitious products, filling pores and refining the pore structure, just as SEM observed that P-10 has no obvious unhydrated particles and a highly dense structure (Figure 7d). As reported by Bertola et al. [21], OPC-induced C-S-H gel densification reduces macropore formation, consistent with our MIP results in the P area.

In the S area, the incorporation of OPC has dual effects on the pore structure: on one hand, the alkalinity introduced by OPC promotes the hydration of ye’elimite into the AFt type, reducing the formation of fluffy AH_3 gel and thus inhibiting macropores larger than 1000 nm; on the other hand, the volume of small pores smaller than 1000 nm increases with higher OPC content, indicating that increased alkalinity refines hydration products and promotes their uniform distribution [50]. Notably, the cumulative pore volume of S-90 is close to that of pure CSA (S-100). Combined with the 32-h duration of the heat release deceleration stage in S-90 (Section 3.1), it is inferred that microcracks in the matrix caused by excessive hydration heat offset the optimizing effect of OPC on the pore structure, corresponding to the mechanism of numerous internal pores observed in S-90 by SEM (Figure 8d).

The interaction between CSA and OPC affects the pore structure by changing the types of hydration products and their packing modes: in the P area, the nucleation-inducing effect of CSA accelerates the generation of C-S-H gel and promotes the ordered growth of ettringite, forming a dense packing structure; in the S area, although the alkalinity introduced by OPC inhibits macropore formation, the inherent rapid hydration and expansion characteristics of CSA still dominate pore structure evolution, resulting in a larger overall pore size in the S area than in the P area. MIP analysis quantifies these differences, further corroborating the conclusion in Section 3.2 that “hydration products in the P area are mainly C-S-H and AFt, while the S area relies on the AFt-type hydration pathway”. This provides experimental evidence at the pore scale for understanding the microstructure-property relationship in the composite system.

The MIP results align with SEM/EDS observations: the refinement of pore structure in the P area stems from the efficient filling of hydration products, while the macropore characteristics in the S area are related to the expansiveness and rapid hydration of CSA. These findings deepen the understanding of the evolution law of the pore structure in the OPC-CSA system and lay a theoretical foundation for improving the durability of materials through mix ratio regulation in the future.

### 3.4. Hydration Kinetic Model and Parameter Analysis

**Figure 11:**
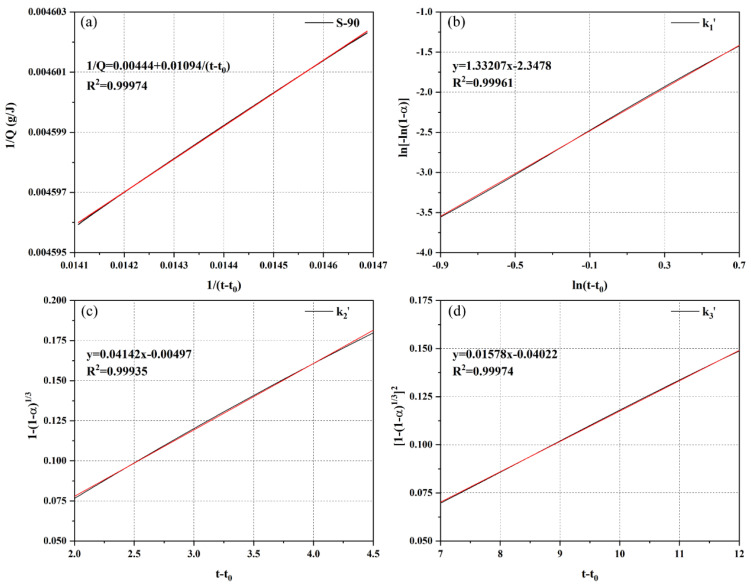
Determination of theoretical maximum release value Q_max_ (**a**), $k_{1}^{\prime }$ (**b**), $k_{2}^{\prime }$ (**c**), and $k_{3}^{\prime }$ (**d**) for S-90 by linear regression.

Figure 11 establishes a quantitative analysis method for the theoretical maximum heat release (Q_max_) and hydration kinetic parameters of S-90 using hydration heat data and linear fitting techniques. Specifically, Figure 11a applies Knudsen’s equation (Equation (7)) to the measured data of S-90. Q_max_ is calculated as the reciprocal of 0.00444, which fully matches the theoretical maximum heat release value of S-90 in Table 5. This method ensures the accuracy and reliability of Q_max_ calculations for samples with different mix ratios through strict linear regression analysis. The fitting results of Knudsen’s equation, correlation coefficients (r^2^), and Q_max_ values for other samples are listed in Table 5, providing basic data support for the kinetic model.

Figure 11b presents the key fitting curve for the NG process. By plotting the double-logarithmic relationship between $ln\left[-\ln\left(1-\alpha \right)\right]$ and $ln\left(t-t_{0}\right)$, the linear transformation of the non-linear equation (Equation (1)) is achieved:

(13) $$ln\left[-\ln\left(1-\alpha \right)\right]=nlnk_{1}^{\prime }+nln\left(t-t_{0}\right)$$

From this, the crystal growth constant *n* and the nucleation growth rate constant $k_{1}^{\prime }$ are directly extracted. Similarly, the parameters $k_{2}^{\prime }$ and $k_{3}^{\prime }$ for the I and D processes are obtained by fitting the linear relationships of Equations (2) and (3), respectively (Figure 11c,d). The degree of hydration α used in all fitting processes is accurately calculated from the measured hydration heat through Equation (8), ensuring the rigor of parameter derivation.

**Figure 12:**
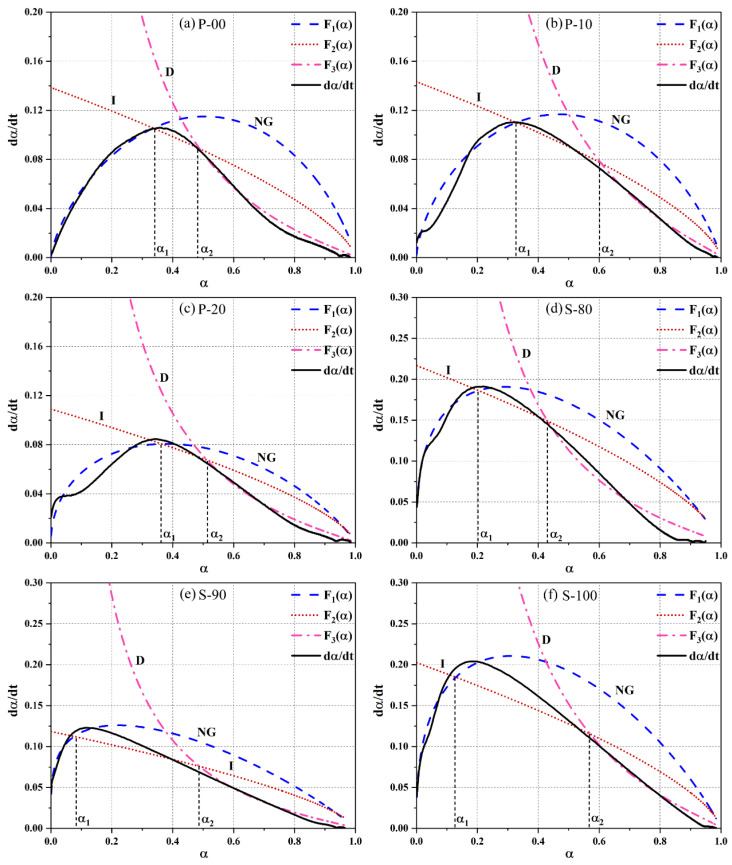
Simulated hydration curves of OPC-CSA.

Figure 12 shows three theoretical rate curves (F₁(α), F_2_(α), and F_3_(α)) derived from the hydration model (Equations (4)–(6)). The hydration degrees α₁ and α_2_ at the intersection points indicate the transitions from the NG to I process and from the I to D process, respectively. These reveal a three-stage dominant mechanism of the hydration process: “nucleation and growth → interfacial reaction → diffusion control”. The simulation deviations in the NG process of the P area and the I process of the S area are attributed to the transitional effects of synergistic hydration between OPC and CSA in the P area and the abnormal decomposition kinetics of ye’elimite under high-alkali conditions in the S area, reflecting the complexity of mineral interactions in the composite system.

The kinetic parameters summarized in Table 6 show that the crystal growth constant n in the P area decreases from 3.3750 (P-00) to 1.9055 (P-20) as the CSA content increases. This indicates that the abundant nuclei provided by ye’elimite hydration cause the NG process to shift from three-dimensional isotropic growth to two-dimensional planar growth. The reaction rates follow a decreasing order of $v_{NG}>v_{I}>v_{D}$, which is due to early autocatalytic reactions (increased nuclei raise $k_{1}^{\prime }$, mid-stage interfacial reactions limited by growth space $k_{2}^{\prime }$ decrease), and late-stage diffusion hindered by a dense product layer ($k_{3}^{\prime }$ is the lowest). In the P area, $k_{1}^{\prime }$ is 1.9–2.3 times higher than $k_{2}^{\prime }$ and 3.8–4.8 times higher than $k_{3}^{\prime }$. In the S area, the ratios of $k_{1}^{\prime }/k_{2}^{\prime }$ and $k_{1}^{\prime }/k_{3}^{\prime }$ are even higher (4.0–10.9 times), reflecting the enhanced dominance of the NG process in the high-CSA environment.

In the P area, the P-10 sample with 10% CSA shows the best kinetic performance. Its $k_{1}^{\prime }$ (0.10768) is 22% higher than that of P-00. This improvement is due to the additional nucleation sites provided by the expansion characteristics of CSA and the rapid nucleation of ettringite promoted by calcium hydroxide (dominated by Reaction 2). When the CSA content increases to 20% (P-20), insufficient calcium hydroxide causes ye’elimite hydration to shift to Reaction 1. The generated $AH_{3}$ gel leads to structural instability, prolonging the NG process and increasing $\alpha_{1}$. Meanwhile, the $k_{2}^{\prime }$ and $k_{3}^{\prime }$ values of P-10 and P-20 are higher than those of P-00, which is consistent with the optimized dense pore structure observed in Section 3.3. This indicates that CSA improves the microstructure by accelerating interfacial reactions and diffusion processes [35].

In the S area, the incorporation of OPC results in significantly lower $k_{1}^{\prime }$ values for S-90 and S-80 compared to the pure CSA sample S-100, reflecting the inhibitory effect of the high-alkali environment on the initial nucleation of ye’elimite. The higher $\alpha_{1}$ value of S-80 is related to the delayed stabilization of hydration products caused by free lime. The decrease in $\alpha_{2}-\alpha_{1}$ with increasing OPC content indicates that excessive OPC accelerates the transition from interfacial reaction to diffusion control by catalyzing the rapid formation of products. Notably, the $k_{3}^{\prime }$ value of S-90 is only 0.07655. Combined with its extremely high $Q_{max}$ (225.23 J/g) and the 32-h heat release deceleration stage described in Section 3.1, this reveals that its hydration process mainly relies on a slow diffusion mechanism, which is kinetically related to the porous structure observed by SEM.

In summary, the Krstulović–Dabić model reveals the hydration kinetic differences in the OPC-CSA system through quantitative parameters: in the P area, CSA accelerates hydration by enhancing nucleation and interfacial reactions; in the S area, OPC inhibits early nucleation and delays the diffusion process through alkalinity regulation. The collaborative analysis of model parameters with heat release behavior and microstructures provides a theoretical basis for optimizing the mix ratio of composite cement systems. The simulation deviations point to directions for further research on the synergistic hydration mechanism of minerals.

## 4. Conclusions

This study systematically reveals the early hydration mechanism of OPC-CSA composite systems through integrated multi-scale characterization and the Krstulović–Dabić kinetic model. The core innovation is the identification of an ‘alkalinity-nucleation coupling mechanism,’ whereby OPC-derived free lime accelerates ye’elimite hydration in CSA-dominated systems, while CSA provides nucleation sites to enhance OPC hydration kinetics. This mechanism tackles the long-standing challenge of optimizing OPC-CSA mix ratios by clarifying the joint regulation of hydration pathways by alkalinity and ion concentration. By categorizing the systems into OPC-dominated (P area) and CSA-dominated (S area), the primary conclusions are as follows:

1. Threshold effects of admixture content on hydration processes. In the P area, the CSA content shows a dual “optimization-inhibition” effect on hydration. A 10% CSA content (P-10) provides nucleation sites through ye’elimite and induces rapid ettringite nucleation via calcium hydroxide. This significantly increases the 3-day cumulative hydration heat (a 63.8% increase compared to pure OPC) and advances the peak heat release rate (by 4.3 h). The nucleation and growth rate constant k_1^’ increases by 22% compared to P-00. However, a 20% CSA content (P-20) inhibits the hydration of OPC silicate minerals due to imbalanced calcium hydroxide supply and excessive sulfate ions, leading to an 18.6% reduction in cumulative hydration heat compared to P-10.
  In the S area, a 10% OPC content (S-90) increases system alkalinity through free lime, promoting the hydration pathway of ye’elimite to shift from the “AH_3 type” (without CH participation) to the “AFt type” (induced by CH). This results in a 71.0% increase in 3-day cumulative hydration heat compared to pure CSA (S-100). But a 20% OPC content (S-80) causes a delayed peak heat release rate and reduced hydration degree due to ion concentration imbalance and steric hindrance between mineral particles.
2. Hydration product formation and mineral interaction mechanisms. In the P area, a 10% CSA content promotes the hydration of OPC’s tricalcium aluminate and belite, leading to increased formation of C-S-H gel and slender ettringite crystals. However, a higher CSA content (20%) causes excessive consumption of calcium hydroxide, shifting ye’elimite hydration to form unstable AH_3_ gel and inhibiting silicate mineral hydration. In the S area, free lime from OPC creates an alkaline environment that facilitates ettringite (AFt) formation during ye’elimite hydration. Yet, the early hydration of OPC’s tricalcium aluminate and belite is suppressed, meaning ye’elimite hydration mainly relies on externally introduced free lime rather than in-situ generated calcium hydroxide. As OPC content increases in the S area, the promotion of ye’elimite hydration toward the AFt type first strengthens and then weakens.
3. Zonal differences in pore structure evolution. In the P area, CSA promotes dense packing of hydration products, resulting in most probable pore sizes below 100 nm. A 10% CSA content significantly reduces cumulative pore volume and refines the pore structure. In the S area, the rapid hydration and expansive properties of CSA lead to most probable pore sizes in the 340–440 nm range, with macropores (2000–8000 nm) present. While OPC incorporation inhibits macropores larger than 1000 nm in the S area, excessive hydration heat in samples like S-90 prevents significant optimization of cumulative pore volume (similar to pure CSA sample S-100), reflecting competitive effects of alkalinity and hydration heat on pore structure.
4. Microstructural characteristics and elemental distribution. At 1-day hydration, P-10 forms a uniform C-S-H gel matrix, which becomes highly dense at 3 days with ettringite exhibiting one-dimensional oriented growth. In contrast, P-20 shows numerous unhydrated OPC particles and interparticle voids due to suppressed hydration. In S-90, ettringite evolves from short rod-shaped to slender crystals with age, indicating alkalinity-promoted AFt-type hydration, but excessive hydration heat leads to a porous internal structure. In S-80, rapidly formed early hydration products hinder ion diffusion, leaving abundant unhydrated particles in pores at 3 days. Dynamic evolution of Al/Si and S/Si atomic ratios from EDS quantitatively validates Si enrichment in the P area (due to C-S-H gel formation) and relative enrichment of aluminum and sulfur in the S area (reflecting differences in ye’elimite hydration pathways).
5. Hydration kinetic model and parameter characteristics. The Krstulović–Dabić model shows that in the P area, CSA increases nucleation sites, causing the nucleation and growth (NG) process to shift from three-dimensional to two-dimensional growth (crystal growth constant n decreases from 3.3750 to 1.9055) and enhances interfacial reaction (I) and diffusion (D) rates (increased $k_{2}^{\prime }$ and $k_{3}^{\prime }$), with P-10 exhibiting optimal kinetic performance. In the S area, OPC suppresses early nucleation of ye’elimite through alkalinity regulation ($k_{1}^{\prime }$ decreases by 38.2% compared to S-100) and prolongs the diffusion process (D). S-90 relies on a slow diffusion mechanism, leading to a 32-h heat release deceleration stage. Model parameters align with microstructural and heat release behaviors, though slight deviations in simulating the S area’s interfacial reaction process require in-situ XRD to track ye’elimite decomposition kinetics.

This study focuses on early hydration (within 3 days), noting that tricalcium aluminate and belite hydration in the S area may delay until 7 days. While the Krstulović–Dabić model captures early kinetics, future research should employ in-situ testing (e.g., XRD) to monitor hydration beyond 3 days, addressing potential delayed ettringite formation—particularly ettringite-to-monosulfate transformation in OPC-dominated systems with excess CSA or secondary ettringite in CSA-dominated systems with high OPC. The dense microstructure of optimized mixes (e.g., P-10) may mitigate delayed expansion by hindering ion diffusion. Additionally, long-term analysis should assess water-to-binder ratio effects (0.25–0.40) on mineral interactions and correlate hydration evolution with macroscopic properties like corrosion resistance and early strength to refine composite design theory.

## Figures and Tables

**Table 1:** Chemical compositions of OPC and CSA (%).

Cement	SiO_2_ (%)	Al_2_O_3_ (%)	Fe_2_O_3_ (%)	CaO (%)	MgO (%)	SO_3_ (%)	Others (%)
OPC	18.60	5.40	2.80	59.50	2.90	4.80	6.00
CSA	11.59	27.67	1.58	45.43	2.52	8.70	2.51

**Table 2:** Mix proportions of cement paste (kg/m^3^).

Series	Number	CSA	OPC	Water	Reducer
P	P-00	0	1500	450	3
	P-10	150	1350	450	3
	P-20	300	1200	450	3
S	S-80	1200	300	450	3
	S-90	1350	150	450	3
	S-100	1500	0	450	3

**Table 3:** Methods and properties.

Object	Method	Property	Parameters
Fresh paste	Isothermal calories calorimetry	Hydration heat	Heat flow and cumulative heat
Hydration product	FT-IR, XRD, TG	Composition, phase, content	/
Hardened paste	MIP	Pore structure	Pore size distribution, total pore volume
Hydration kinetics	Krstulović–Dabić model	Hydration mechanism	$k_{1}^{\prime },\;k_{2}^{\prime },\;k_{3}^{\prime }$
Hardened paste	SEM	Microstructure analysis and elemental distribution	/
Hardened paste	EDS	Atomic ratio of Al and S to Si	Atomic concentration percentage of Al, S, Si

**Table 4:** Hydration products calculated by TG data.

Area	Number	Weight Loss/wt%				Content of Products/wt%		
		AFt	AH_3_	Ca(OH)_2_	AFm	AFt	AH_3_	Ca(OH)_2_
P	P-00	4.47	-	3.08	2.64	12.76	-	12.65
	P-10	4.79	-	2.35	3.19	13.67	-	9.68
	P-20	4.99	-	0.78	2.95	14.26	-	3.19
S	S-80	8.67	1.3	-	3.64	24.78	3.75	-
	S-90	8.36	1.36	-	3.27	23.89	3.93	-
	S-100	8.52	1.36	-	-	24.36	3.93	-

**Table 5:** Results of Knudsen equations and Qmax.

Area	Number	Knudsen Equations	r^2^	Q_max_
P	P-00	1/Q = 0.00843 + 0.0169/(t − t_0_)	0.99872	118.62
	P-10	1/Q = 0.00518 + 0.00387/(t − t_0_)	0.99833	193.05
	P-20	1/Q = 0.00634 + 0.00679/(t − t_0_)	0.99943	157.73
S	S-80	1/Q = 0.0097 + 0.03779/(t − t_0_)	0.99100	103.09
	S-90	1/Q = 0.00444 + 0.01094/(t − t_0_)	0.99974	225.23
	S-100	1/Q = 0.00773 + 0.00918/(t − t_0_)	0.99887	129.37

**Table 6:** Hydration kinetic parameters of OPC-CSA.

Area	Number	n	$k_{1}^{\prime }$	$k_{2}^{\prime }$	$k_{3}^{\prime }$	α_1_	α_2_	α_2_ − α_1_
P	P-00	3.3750	0.08825	0.04620	0.01851	0.33593	0.48862	0.15269
	P-10	2.7286	0.10768	0.04777	0.02551	0.32749	0.60618	0.27869
	P-20	1.9055	0.09705	0.04633	0.02548	0.36307	0.53265	0.16958
S	S-80	1.5165	0.25515	0.06224	0.02474	0.20380	0.43077	0.22697
	S-90	1.3321	0.17161	0.04142	0.01578	0.07655	0.48761	0.41106
	S-100	1.5860	0.27774	0.06751	0.03316	0.12919	0.57065	0.44146

## Data Availability

The original contributions presented in this study are included in the article. Further inquiries can be directed to the corresponding author.

## References

[1] Cho S., Suh H., Kim G., Liu J., Li P., Bae S. (2024). Microstructural phase evolution and strength development of low-lime calcium silicate cement (CSC) paste incorporating ordinary Portland cement under an accelerated carbonation curing environment. Constr. Build. Mater..

[2] Oke J.A., Abuel-Naga H., Leong E.C. (2024). De-carbonizing in construction using supplementary cementitious materials and accelerated carbonation technique—A review. Int. J. Geotech. Eng..

[3] de Melo J.V.S., Trichês G. (2018). Study of the influence of nano-TiO2 on the properties of portland cement concrete for application on road surfaces. Road Mater. Pavement Des..

[4] Shen X., Guo S., Li W., Lu C., Zhang K., Wang M., Wen Z. (2023). Research Status on Hydration and Properties of Low-Heat Portland Cement. Bull. Chin. Ceram. Soc..

[5] Liu S., Wei L., Zhou S., Zhao S., Guan X., Wang L. (2014). Research Development of High-strength Low-calcium Portland Cement. Bull. Chin. Ceram. Soc..

[6] Aliabdo A.A., Elmoaty A.E.M.A., Mohamed M.F. (2017). Permeability indices and corrosion resistance of geopolymer and portland cement concretes. Mag. Concr. Res..

[7] Chen J.J., Sorelli L., Vandamme M., Ulm F.-J., Chanvillard G. (2010). A coupled nanoindentation/SEM-EDS study on low water/cement ratio portland cement paste: Evidence for C–S–H/Ca(OH)2 nanocomposites. J. Am. Ceram. Soc..

[8] Comin-Chiaramonti L., Cavalleri G., Sbaizero O., Comin-Chiaramonti P. (2009). Crystallochemical comparison between portland cements and mineral trioxide aggregate (MTA). J. Appl. Biomater. Biomech..

[9] Zhang H., Su Y., Yang Z., Zhang X. (2018). Early hydration process of the cement–lime system. Ce/Papers.

[10] Lai-Guo W., Ling-Chao L.U., Xin C. (2004). Development of composite cement materials on portland and sulphoaluminate minerals. J. Shandong Inst. Build. Mater..

[11] Tang X., Zhan S., Xu Q., He K. (2023). Mechanical Performance and Chloride Penetration of Calcium Sulfoaluminate Concrete in Marine Tidal Zone. Materials.

[12] Lu L., Wang S., Cheng X. (2012). Effect of admixture on sulfate resistance of alite-barium calcium sulphoaluminate cement mortar. Procedia Eng..

[13] Li J., Huang J., Yang M., Wang L., Ma J. (2017). Study on Workability and Early Compression Strength of OPC-SAC Concrete. Constr. Technol..

[14] Huo G., Jiang X., Sun X., Li H., Shi H. (2024). Performance of high-belite calcium sulfoaluminate cement subjected to hydrochloric acid and sulfuric acid. Front. Mater..

[15] Zhang P., Li Y., Wang W., Wen D., Xiao W. (2018). Modification of Sulpho—Aluminate Cement and Its Application in Electrical Ceramic Cement Compo. Insul. Surge Arresters.

[16] Liu X.C., Li Y.J. (1998). Research on the Composite Properties of Alite-Sulphoaluminate Cement and Portland Cement. Cement.

[17] Wang F.S., Yang H.Y. (1997). Experimental Research on Composite Sulphoaluminate Cement. Shandong Build. Mater..

[18] Wang Z., Lan W., Jia Z., Lin M., Li D. (2024). Research on the Mechanical Properties of Composite Grouting Materials Based on Ordinary Portland–Sulphoaluminate Cement. Buildings.

[19] Mehta P.K., Klein A. (1966). Investigations on the Hydration Products in the System 4 CAO-3AL203-SO3-CASO4-CAO-H20.

[20] Gastaldi D., Bertola F., Irico S., Paul G., Canonico F. (2021). Hydration behavior of cements with reduced clinker factor in mixture with sulfoaluminate binder. Cem. Concr. Res..

[21] Bertola F., Gastaldi D., Irico S., Paul G., Canonico F. (2022). Influence of the amount of calcium sulfate on physical/mineralogical properties and carbonation resistance of CSA-based cements. Cem. Concr. Res..

[22] Gastaldi D., Canonico F., Capelli L., Bianchi M., Telesca A., Valenti G.L. Hydraulic behaviour of calcium sulfoaluminate cement alone and in mixture with Portland cement. Proceedings of the 13th International Congress on the Chemistry of Cement.

[23] Marchi M., Costa U. Influence of the calcium sulphate and w/c ratio on the hydration of calcium sulfoaluminate cement. Proceedings of the 13th International Congress on the Chemistry of Cement.

[24] Kuzel H.-J. (1996). Initial hydration reactions and mechanisms of delayed ettringite formation in portland cements. Cem. Concr. Compos..

[25] Cui H., Wang C., Zhang G., Zhang K., Fu X., Bai Y., Zheng Y., Qi Y., Liu Z. (2024). Preparation and hydration mechanism of composite cementitious materials based on iron and steel solid waste. Alex. Eng. J..

[26] Chang J., Zhang Y., Shang X., Zhao J., Yu X. (2017). Effects of amorphous AH3 phase on mechanical properties and hydration process of C4A3S¯-CS¯H2-CH-H2O system. Constr. Build. Mater..

[27] Pelletier L., Winnefeld F., Lothenbach B. (2010). The ternary system portland cement–calcium sulphoaluminate clinker–anhydrite: Hydration mechanism and mortar properties. Cem. Concr. Compos..

[28] Trauchessec R., Mechling J.-M., Lecomte A., Roux A., Le Rolland B. (2015). Hydration of ordinary portland cement and calcium sulfoaluminate cement blends. Cem. Concr. Compos..

[29] Zhang J., Li G., Ye W., Chang Y., Liu Q., Song Z. (2018). Effects of ordinary portland cement on the early properties and hydration of calcium sulfoaluminate cement. Constr. Build. Mater..

[30] Kondo R., Kodama M. (1967). On the hydration kinetics of cement. Semento Gijutsu Nenpo.

[31] Johnson W.A. (1939). Reaction kinetics in process of nucleation and growth. Trans. Trans. Am. Inst. Min. Metall. Eng..

[32] Krstulović R., Dabić P. (2000). A conceptual model of the cement hydration process. Cem. Concr. Res..

[33] Zhang J., Wang X., Jin B., Zhang X., Li Z., Guan X. (2021). Effect of superplasticizers on hydration kinetics of ultrafine sulfoaluminate cement-based grouting material. Thermochim. Acta.

[34] Iqbal M.A., Sahar U.U., Bahrami A., Yaseen N., Siddique I. (2024). Development of Sugarcane Bagasse Ash Blended Cementitious Composites Reinforced with Carbon Nanotubes and Polypropylene Fibers. J. Compos. Sci..

[35] Meng T., Hong Y., Wei H., Xu Q. (2019). Effect of nano-SiO_2_ with different particle size on the hydration kinetics of cement. Thermochim. Acta.

[36] Li Y., Guo Y., Lyu Z., Wei X. (2021). Investigation of the effect of waterborne epoxy resins on the hydration kinetics and performance of cement blends. Constr. Build. Mater..

[37] Yang M., Chen L., Lai J., Osman A.I., Farghali M., Rooney D.W., Yap P.-S. (2024). Advancing environmental sustainability in construction through innovative low-carbon, high-performance cement-based composites: A review. Mater. Today Sustain..

[38] Zhang G., Xia H., Wang H., Song L., Niu Y., Cao D., Chen H. (2023). Early hydration characteristics and kinetics model of cement pastes containing internal curing materials with different absorption behaviors. Constr. Build. Mater..

[39] Knudsen T. (1980). On particle size distribution in cement hydration. Proceedings of the International Congress on the Chemistry of Cement Paris.

[40] Martinelli E., Koenders E.A.B., Caggiano A. (2013). A numerical recipe for modelling hydration and heat flow in hardening concrete. Cem. Concr. Compos..

[41] Pepe M., Lima C., Martinelli E. (2020). Early-age properties of concrete based on numerical hydration modelling: A parametric analysis. Materials.

[42] Allen A.J., Thomas J.J. (2007). Analysis of C–S–H gel and cement paste by small-angle neutron scattering. Cem. Concr. Res..

[43] Li D.D. (1984). Infrared spectroscopic study of sulfoaluminate cement. J. Chin. Ceram. Soc..

[44] Pelletier-Chaignat L., Winnefeld F., Lothenbach B., Müller C.J. (2012). Beneficial use of limestone filler with calcium sulphoaluminate cement. Constr. Build. Mater..

[45] Zajac M., Hoock S., Stabler C., Haha M.B. (2017). Effect of hydration kinetics on properties of compositionally similar binders. Cem. Concr. Res..

[46] Li G., He T., Hu D., Shi C. (2012). Effects of two retarders on the fluidity of pastes plasticized with aminosulfonic acid-based superplasticizers. Constr. Build. Mater..

[47] Scrivener K., Ouzia A., Juilland P., Mohamed A.K. (2019). Advances in understanding cement hydration mechanisms. Cem. Concr. Res..

[48] Goetz-Neunhoeffer F., Neubauer J., Schwesig P. (2006). Mineralogical characteristics of ettringites synthesized from solutions and suspensions. Cem. Concr. Res..

[49] Chen I.A., Hargis C.W., Juenger M.C.G. (2012). Understanding expansion in calcium sulfoaluminate–belite cements. Cem. Concr. Res..

[50] Zhang Y., Wang S., He S., Hao X. (2023). Analysis of factors influencing the temperature field variation in mass concrete during hydration heat release. Case Stud. Therm. Eng..

[51] Li P., Gao X., Wang K., Tam V.W.Y., Li W. (2020). Hydration mechanism and early frost resistance of calcium sulfoaluminate cement concrete. Constr. Build. Mater..

[52] Li P., Li W., Yu T., Qu F., Tam V.W.Y. (2020). Investigation on early-age hydration, mechanical properties and microstructure of seawater sea sand cement mortar. Constr. Build. Mater..

[53] Gomes S.D.C., Zhou J.L., Li W., Qu F. (2020). Recycling of raw water treatment sludge in cementitious composites: Effects on heat evolution, compressive strength and microstructure. Resour. Conserv. Recycl..

[54] Odler I., Colán-Subauste J. (1999). Investigations on cement expansion associated with ettringite formation. Cem. Concr. Res..

